# Longitudinal analysis to characterize classes and subclasses of antibody responses to recombinant receptor-binding protein (RBD) of SARS-CoV-2 in COVID-19 patients in Thailand

**DOI:** 10.1371/journal.pone.0255796

**Published:** 2021-08-10

**Authors:** Sarunporn Tandhavanant, Sirikamon Koosakunirand, Taniya Kaewarpai, Watcharapong Piyaphanee, Pornsawan Leaungwutiwong, Viravarn Luvira, Narisara Chantratita

**Affiliations:** 1 Department of Microbiology and Immunology, Faculty of Tropical Medicine, Mahidol University, Bangkok, Thailand; 2 Department of Clinical Tropical Medicine, Faculty of Tropical Medicine, Mahidol University, Bangkok, Thailand; 3 Mahidol-Oxford Tropical Medicine Research Unit, Faculty of Tropical Medicine, Mahidol University, Bangkok, Thailand; Emory University School of Medicine, UNITED STATES

## Abstract

Serological assays to detect antibodies against severe acute respiratory syndrome coronavirus 2 (SARS-CoV-2) might contribute to confirming the suspected coronavirus disease 2019 (COVID-19) in patients not detected with molecular assays. Human antibodies that target the host angiotensin-converting enzyme 2-binding domain of the viral spike protein are a target for serodiagnosis and therapeutics. This study aimed to characterize the classes and subclasses of antibody responses to a recombinant receptor-binding protein (RBD) of SARS-CoV-2 in COVID-19 patients and investigated the reactivity of these antibodies in patients with other tropical infections and healthy individuals in Thailand. ELISAs for IgM, IgA, IgG and IgG subclasses based on RBD antigen were developed and tested with time series of 27 serum samples from 15 patients with COVID-19 and 60 samples from pre-COVID-19 outbreaks including acute dengue fever, murine typhus, influenza, leptospirosis and healthy individuals. Both RBD-specific IgA and IgG were detected in only 21% of the COVID-19 patients in the acute phase. The median IgA and IgG levels were significantly higher in the convalescent serum sample compared to the acute serum sample (*P* < 0.05). We observed the highest correlation between levels of IgG and IgA (rho = 0. 92). IgG1 and IgG3 were the major IgG subclasses detected in SARS-CoV-2 infection. Only acute IgG3 level was negatively associated with viral detection based on RT-PCR of *ORF1ab* gene (rho = -0.57). The median IgA and IgG levels in convalescence sera of COVID-19 patients were significantly higher than healthy individuals and convalescent sera of other febrile infectious patients. The analyses of antibody classes and subclasses provide insights into human immune responses against SARS-CoV-2 during natural infection and interpretation of antibody assays.

## Introduction

The coronavirus disease 2019 (COVID-19) is a pandemic infectious disease caused by a novel coronavirus known as severe acute respiratory syndrome coronavirus 2 (SARS-CoV-2). Structurally, SARS-CoV-2 contains four structural proteins, including spike (S), nucleocapsid (N), membrane (M) and envelope (E) proteins [1]. The SARS-CoV-2 S protein consists of the S1 subunit (14–685 residues) and the S2 subunit (686–1273 residues) [2]. The S1 subunit has a receptor-binding domain (RBD) that recognizes and binds to the human angiotensin-converting enzyme 2 (ACE2). The SARS-CoV-2 and severe acute respiratory syndrome coronavirus (SARS-CoV) RBD are ~73%–76% similar in genome sequence [3]. The RBD region of SARS-CoV is an immunogenic antigen and can elicit neutralizing antibodies in the patients infected with these coronaviruses [4]. The receptor-binding motif (RBM), a portion of RBD making direct contacts with ACE2, is used as an antigen and an important target for antibody detection in SARS-CoV patients [5, 6].

Similar to SARS-CoV, the RBD of SARS-CoV-2 is an immunodominant viral glycoprotein that mediates binding to human ACE2 receptor and induces a high amount of specific and neutralizing antibodies in COVID-19 patients [7, 8]. Neutralizing antibodies against RBD have shown protection against SARS-CoV-2 infection in ACE2-expressing HEK293T cells [7, 8]. The RBD of SARS-CoV-2 is, therefore, a promising antigen for serodiagnosis [9, 10] and a potential antigen for prophylactic and therapeutic effects in human COVID-19 [11–13].

The current standard assay for COVID-19 diagnosis is the molecular detection of viral RNA, however, the rapid evolution of the virus may lead to an increased false-negative rate by the molecular detection method. The antibody-based immunological assay is an alternative to the RNA detection method in the diagnosis of late presentations of COVID-19 [14, 15]. The serological tests specific to SARS-CoV-2 may provide additional information, not only as affordable diagnostic tools but the data may be useful for an epidemiological study. Moreover, a greater understanding of the antibody response is important for the development of a vaccine and guiding control measures for the government.

The antibody has various classes and subclasses which are timely produced from plasma cells in different sites and perform diverse functions. IgM and IgA are theoretically produced during the first week of infection while IgG is detected later. IgA is mainly secreted from the mucosal tissue and prevents infection in the respiratory and gastrointestinal tract. IgG1 and IgG3 subclasses have high-affinity binding to Fc receptors, leading to enhanced opsonization and phagocytosis [16]. IgG2 subclass is a major antibody that is responsive to carbohydrate antigens. IgG4 subclass plays a critical role in allergy as a blocking antibody [17]. IgG3 subclass has an important role in viral infections where the antibody level is associated with viral neutralization and clearance [18, 19]. Therefore, the detection of immunoglobulin classes and subclasses can be utilized for understanding humoral immune responses during infection.

Antibody-based enzyme-linked immunosorbent assay (ELISA) is a powerful immunoassay for massive serological testing. In a study conducted in Wuhan Hospital (Wuhan, China), serum IgM and IgA antibodies against SARS-CoV-2 were detectable 3–6 days and serum IgG antibody 10–18 days post clinical symptoms onset in COVID-19 patients by ELISA based on the viral nucleocapsid protein [20]. However, the timing of blood collection, type of antibody, antigen targets and immune responses in different populations can contribute to the accuracy of the test.

As of March 03, 2021, the total number of confirmed COVID-19 cases in Thailand has reached 26,108, with 84 deaths (https://covid19.who.int/region/searo/country/th). Most of the patients with COVID-19 who visited hospitals showed non-specific symptoms including fever, cough and sore throat similar to those patients with other tropical infections [21]. The most common tropical diseases that cause a major acute undifferentiated fever in Thailand are dengue fever, murine typhus, seasonal influenza and leptospirosis [22]. It is important to evaluate the sensitivity and the specificity of these tropical diseases to implement a serological test for COVID-19 in tropical countries.

In our study, we determined and characterized the antibody classes (IgM, IgA and IgG) and subclasses (IgG1, IgG2, IgG3 and IgG4) to RBD of SARS-CoV-2 with time series of 27 serum samples from 15 patients with COVID-19. We further investigated the association of antibody classes and subclasses with viral detection and evaluated the diagnostic role of these antibodies in Thailand.

## Materials and methods

### Serum samples

For this study, we included 27 serum specimens from 15 COVID-19 patients admitted to the Hospital for Tropical Diseases, Faculty of Tropical Medicine, Mahidol University, Bangkok, Thailand during the first COVID-19 outbreak in Thailand between February and April 2020. All the cases were diagnosed for COVID-19 and confirmed to be infected with SARS-CoV-2 by real-time reverse transcription PCR (RT-PCR) in the nasopharyngeal swab and throat swab (NPS/TS) using a novel coronavirus 2019-nCoV RNA detection kit (DaAn Gene Co., Ltd., Guangdong, China) as described [23]. Day 0 was considered as the day when COVID-19 patients had an initial illness or were diagnosed with the infection. Serum samples were collected 1–3 times from each COVID-19 patient between days -2 and 35. The acute serum samples were collected from patients within the first week of diagnosis (≤ 7 days). The convalescent serum samples were collected from patients after first week of diagnosis (> 7 days).

Serum samples from 20 healthy individuals were collected at Udon Thani Hospital, Udon Thani in Northeast Thailand between August 2018 and August 2019 before the COVID-19 outbreak and included in this study. Healthy blood donor’s criteria include age ≥18 years and able to understand and provide informed consent. Exclusion criteria included pregnancy or delivery in the past nine months, weight less than 40 kg or greater than 136 kg, recent illness, any chronic medical condition or medications and any organ failure, any immune system deficiency, vaccination within the past six weeks, use of any immune modifying agents or any anti-inflammatory medications or biologic drugs in the past week, infectious symptoms in the past two weeks, vigorous exercise in the past 24 hours, or alcohol use in the past 24 hours [24].

### Serum samples from patients with other infections

The convalescent serum samples were from forty patients diagnosed with dengue fever (N = 10), leptospirosis (N = 10), murine typhus (N = 10) and influenza (N = 10) were used to test the specificity of the ELISAs. The patients were enrolled at the Hospital for Tropical Diseases, Bangkok, Thailand during 2013–2015 [25]. The patients were identified with the infections with dengue virus, *Leptospira*, *Rickettsia typhi* and influenza virus by molecular detection and/or serological assay as described [25].

### Ethical approval

This study was reviewed and approved by the Ethics Committee of the Faculty of Tropical Medicine, Mahidol University (MUTM 2020-043-01). The informed consent was waived because we used previously collected samples from the approved protocols.

### Development of ELISA to detect anti-SARS-CoV-2 S RBD antibodies in serum samples

The ELISA test was developed to detect anti-SARS-CoV-2 S RBD antibodies using GenScript (Z03483) recombinant SARS-CoV-2 S RBD. The concentrations of RBD antigen, detection antibodies and serum samples were optimized. The optimal RBD antigen concentrations were 4μg/ml for IgM and IgA ELISAs and 2μg/ml for IgG and IgG subclass ELISAs. The detection antibodies, horseradish peroxidase (HRP)-conjugated anti-human IgM (DAKO, Copenhagen, Denmark), IgA (Invitrogen, MD, USA), IgG (DAKO) and IgG subclasses (Invitrogen) were used at dilutions of 1:1000, 1:2000, 1:4000 and 1:100, respectively. The optimal serum dilution was 1:100.

50 μl of RBD antigen in 0.05 M sodium carbonate buffer (pH 9.6) was coated on a 96-well ELISA plate (Nunc MaxiSorp U-bottom 96-Well plates; Thermo Scientific, Denmark) and incubated overnight at 4°C. Sample wells were washed with 300 μl of phosphate-buffered saline (PBS) containing 0.05% Tween-20 for four times using a Hydrospeed washer (TECAN, Männedorf, Switzerland) and blocked with 200 μl of 5% skim milk in PBS at 37°C for 2 h. 50 μl of diluted serum samples at final dilution of 1:100 (in 1% bovine serum albumin and 0.05% Tween-20 in PBS) were then added to antigen-coated and uncoated wells and incubated at room temperature for 1 h. Wells were then washed for the second time as described above and incubated with 50 μl of optimized dilution of HRP-conjugated anti-human immunoglobulins at room temperature for 1 h. Wells were washed again and the colorimetric signal was developed by addition of 50 μl 3, 3′,5,5′-tetramethylbenzidine (TMB) with peroxidase (Novex, Liftechnologies, MD, USA). The reaction was stopped after incubating at room temperature for 30 min by the addition of 50 μl 1 N HCl. The absorbance was measured at an optical density (OD) of 450 nm with a Sunrise^TM^ microplate reader (TECAN).

The positive control was a RT-PCR-confirmed COVID-19 patient serum sample. Negative control was the pooled healthy donors’ sera (N = 5). The OD value of a blank which contained only an assay diluent was subtracted from all the OD values of test samples. The OD values of individual samples in uncoated wells were measured. The antibody levels were determined by dividing the sample OD in coated well by the OD of the same serum sample in uncoated well. The ratio value of antibodies level ≥ 1.05 was considered as a presence of specific antibodies against RDB antigen in the patients. All samples were performed ELISA in duplicates.

### Statistical analysis

Statistical analysis was performed using GraphPad Prism version 7.0 (GraphPad Software Inc, La Jolla, CA). Wilcoxon signed-rank test was used for comparing the antibody levels between admission and recovery periods. Mann-Whitney U test was used for testing the difference of medians of non-normally distributed data. Spearman’s rank correlation was used to determine the pairwise correlation coefficient (rho) between pairs of antibody classes and subclasses. Correlations were defined as very high correlation (0.9 to 1.0 and −0.9 to −1.0), high correlation (0.7 to 0.9 and −0.7 to −0.9), moderate correlation (0.5 to 0.7 and −0.5 to −0.7), low correlation (0.3 to 0.5 and −0.3 to −0.5) and negligible correlation (0.0 to 0.3 and 0.0 to −0.3) [26]. Discrimination of acute and convalescent serum of COVID-19 patients was performed by quantifying the area under the receiver operating characteristic (ROC) curve for each antibody. *P* values < 0.05 were considered statistically significant. Sensitivity, specificity, positive and negative predictive values were calculated as follows: Sensitivity = True positive / (true positive + false negative) × 100; Specificity = True negative / (true negative + false positive) ×100; Positive predictive value = true positive / (true positive + false positive) ×100; Negative predictive value = true negative / (true negative + false negative) ×100.

## Results

### Characteristic of COVID-19 patients

Demographic and clinical characteristics of COVID-19 patients are shown in Table 1. Of fifteen COVID-19 patients included in the study, eight (53%) were male and seven (47%) were female. The median age of all patients was 30 years (interquartile range (IQR) = 27–45). Pneumonia was found in four patients whereas the other 11 patients with mild respiratory symptom were classified as mild cases. Two patients with pneumonia had underlying diseases. Both of them had dyslipidemia. Additionally, one of them had diabetes and hypertension.

**Table 1:** Patient demographics and clinical characteristics.

Patient	Acute serum (day)	Convalescent (day)		Age	Sex	Underlying diseases	Symptom	Ct value *ORF1ab* gene
		Serum 1	Serum 2					
**P1** ^a^	-2	9	NA	56	F	None	pneumonia	NA
**P2**	6	29	NA	45	M	None	mild	14.93
**P3**	7	NA	NA	34	M	None	mild	NA
**P4**	4	12	NA	36	F	None	mild	20.82
**P5**	6	23	NA	28	F	None	mild	37.7
**P6** ^a^	2	21	NA	23	F	None	mild	24.2
**P7**	NA	10	NA	24	F	None	mild	14.41
**P8**	6	24	35	36	M	None	mild	32.12
**P9**	3	18	31	68	M	DM, HT, DLP	pneumonia	19.32
**P10**	1	21	NA	28	M	None	mild	28.83
**P11**	4	NA	NA	25	F	Alcoholism	mild	NA
**P12**	2	21	NA	30	M	None	pneumonia	26.19
**P13**	5	20	NA	47	M	DLP	pneumonia	32.19
**P14**	3	NA	NA	27	F	Depression	mild	NA
**P15**	7	NA	NA	27	M	Substance abuse	mild	34.79

The acute serum samples were collected from patients within the first week of diagnosis (≤ 7 days). The convalescent serum samples were collected from patients after first week of diagnosis (> 7 days).

^a^, the patient who was asymptomatic at the diagnosis date.

DM: Diabetes mellitus; HT: Hypertension; DLP: Dyslipidemia; NA: Not available.

### Longitudinal analyses of IgM, IgA and IgG levels in COVID-19 patients

**Fig 1:**
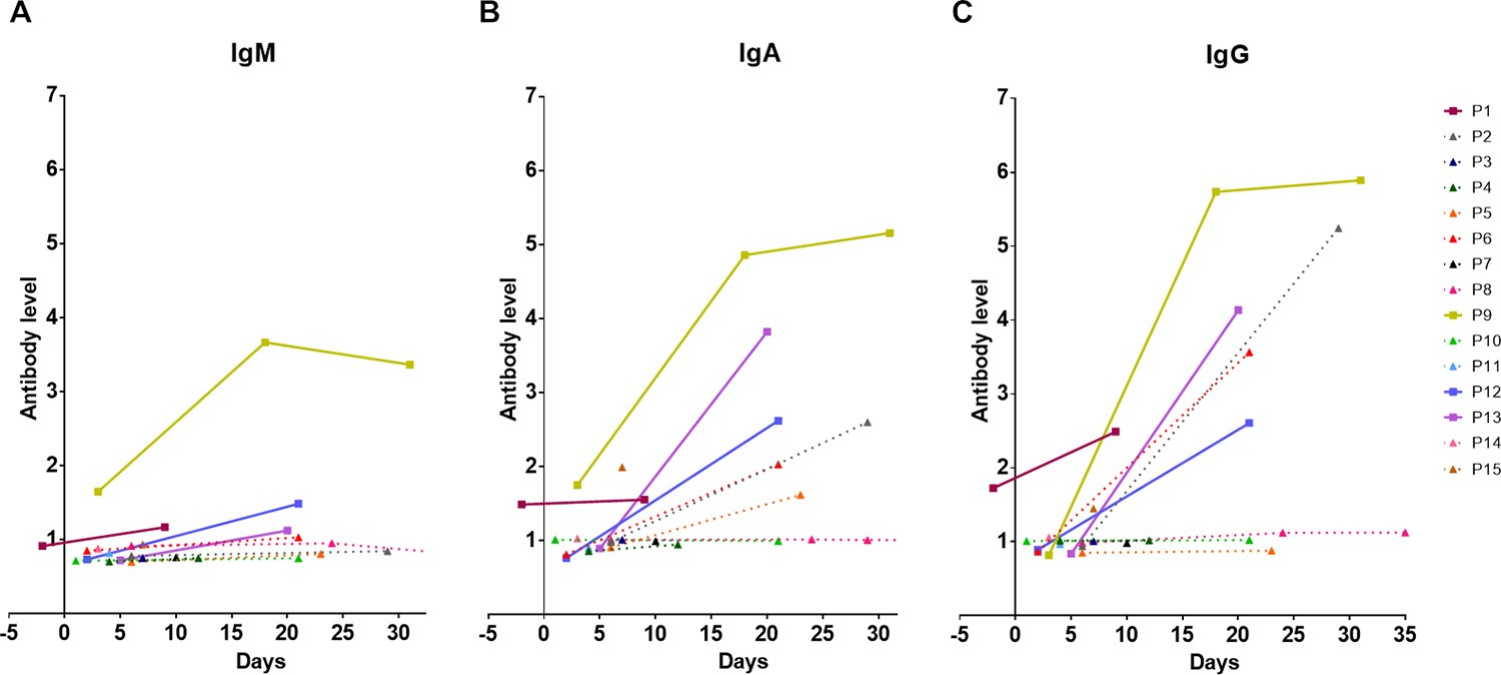
Longitudinal analysis of classes of antibody responses to RBD in COVID-19 patients. Dynamic changes in RBD-specific IgM (A), IgA (B) and IgG (C) antibody response in individual COVID-19 patients (N = 15). Data are plotted by antibody response calculated as the ratio between OD of uncoated and coated antigen well of an individual sample. Each line represents an individual subject. Solid line and square symbol represent pneumonia cases. Dot line and triangle symbol represent mild cases.

A total of 27 serum samples from 15 patients were analyzed with ELISAs measuring IgM, IgA and IgG specific for SARS-CoV-2 RBD in COVID-19 patients (Fig 1). The acute serum samples were collected within the first week of admission from 14 patients with a median time of 4 days (IQR = 2–6). The convalescent serum from 11 patients was obtained during the treatment with a median time of 21 days (IQR = 18–24). We observed that 93% (13 of 14) of COVID-19 patients had IgM levels lower than the diagnostic threshold value of 1.05 at the acute phase with a median IgM level of 0.80 (IQR = 0.72–0.92, Fig 1A and S1 Table). We also observed low levels of IgA and IgG antibodies targeting RBD antigen at the first week of admission with a median level of 0.99 (IQR = 0.88–1.02) for IgA and a median level of 0.96 (IQR = 0.86–1.00) for IgG (Fig 1B, 1C and S1 Table). Furthermore, IgA and IgG levels were higher than IgM in the acute serum sample of COVID-19 patients (*P* = 0.002 and *P* = 0.016, respectively).

We then compared the antibody levels reacting against the RBD antigen in convalescent serum samples collected between admission and recovery periods in 10 patients who had paired sera. The IgM levels were slightly increased in the convalescent period with a median level of 0.99 (IQR = 0.81–1.17) compared to the levels at the admission with a median level of 0.76 (IQR = 0.72–0.91; *P* = 0.005). Four of ten patients (P1, P9, P12 and P13) showed increased IgM levels at the recovery period while other patients did not show increased IgM levels in the convalescent period until 30 days. Three patients (P9, P12 and P13) showed a 1.5-fold increased IgM level (Fig 1A and S1 Table).

For IgA and IgG responses, COVID-19 patients showed markedly increased levels during the recovery period (Fig 1B, 1C and S1 Table). The concentration of IgA was significantly higher in the convalescent serum compared to the acute serum sample (median level of 1.82, IQR 1.00–2.61 versus the median level of 0.94, IQR 0.85–1.00; *P* = 0.011). Similar to IgA, IgG levels significantly increased after the second week of illness (median level of 2.54, IQR = 1.01–4.12 for convalescent serum versus the median level of 0.91, IQR = 0.84–1.00 for acute serum; *P* = 0.005). Half of ten patients showed a more than 1.5-fold increase in both IgA and IgG levels during the convalescent period. One patient (P8) did not develop a specific IgA antibody and one patient (P5) did not develop IgG antibody in the convalescent period. The other two patients (P4 and P10) showed neither IgA nor IgG antibodies against RBD antigens after the second week of their illness.

The correlation between RBD-specific IgM, IgA and IgG levels in 27 serum samples from 15 COVID-19 patients were analyzed (S1 Fig). All correlations were statistically significant. We found a very high correlation between IgG and IgA levels (rho = 0.92, *P* < 0.001) and high correlation between IgM and IgA levels (rho = 0.87, *P* < 0.001) and between IgG and IgM levels (rho = 0.74, *P* < 0.001) (S1A–S1C Fig).

### Association of demographics and clinical characteristics of COVID-19 patients with SARS-CoV-2 antibody response

The results of the analyses of the association of demographics and clinical characteristics of COVID-19 patients with IgM, IgA and IgG are shown in S2 Table. The SARS-CoV-2 specific IgM, IgA and IgG antibodies were detected in 4, 8 and 9 patients, respectively. The median of earliest date of IgM, IgA and IgG antibody detection were 14.5 days (IQR = 6–20.5), 20.5 days (IQR = 5–22) and 20 days (IQR = 3–21), respectively. None of the SARS-CoV-2 specific antibodies either in acute or convalescent samples were detected in five patients (P3, P4, P7, P10 and P11) over time. Older age and the COVID-19 patients with pneumonia cases were associated with the IgM detection in the COVID-19 patients (*P* < 0.05; S2 Table). COVID-19 patients who had pneumonia symptoms developed IgA earlier than the mild cases (11.5 days (IQR = 0.5–20.5) for patients with pneumonia versus 22 days (IQR = 14–26) for mild cases) (Table 1 and S1 Table). Sex was not associated with detections of RBD-specific antibodies (*P* > 0.05; S2 Table). We did not analyze the association of antibody response with underlying disease because the number of these cases was low (1–2 cases).

### Longitudinal analysis of IgG subclasses of antibody responses in COVID-19 patients

**Fig 2:**
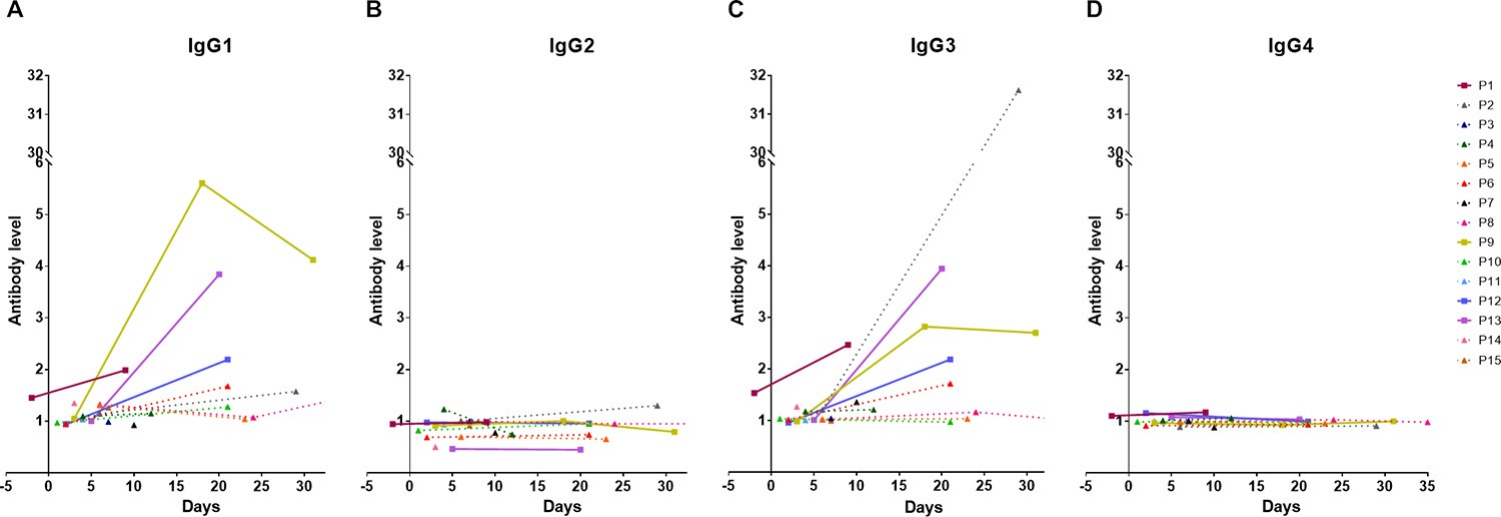
Longitudinal analysis of IgG subclasses of antibody responses to RBD in COVID-19 patients. Dynamic changes in RBD-specific IgG subclasses including IgG1 (A), IgG2 (B), IgG3 (C) and IgG4 (D) antibody response in individual COVID-19 patients (N = 15). Data are plotted by antibody response calculated as the ratio between OD of uncoated and coated antigen wells of an individual sample. Each line represents an individual subject. Solid line and square symbol represent pneumonia cases. Dot line and triangle symbol represent mild cases.

We evaluated the levels of IgG subclasses in 27 serum samples from 15 COVID-19 patients (Fig 2). Higher levels of IgG1 (median level = 1.08, IQR = 0.99–1.31) and IgG3 (median level = 1.03, IQR = 1.01–1.18) were observed in our acute samples (Fig 2A, 2C and S3 Table). IgG1 and IgG3 were detected in 57.14% and 28.57% of COVID-19 patients since the first week of illness. Low detection was found for IgG2 (7.14%) and IgG4 (21.43%) since the first week of illness and the median level for IgG2 and IgG4 was 0.93 (IQR = 0.69–1.0) and 0.99 (IQR = 0.98–1.01), respectively (Fig 2B, 2D and S3 Table). The levels of IgG1 and IgG3 were significantly higher than the level of IgG2 and IgG4 during acute phase of infection (IgG1 versus IgG2, *P* = 0.004; IgG1 versus IgG4, *P* = 0.022; IgG2 versus IgG3, *P* = 0.004; IgG3 versus IgG4, *P* = 0.035).

We further evaluated the antibody response at the admission and convalescent period in 10 COVID-19 patients (S3 Table). IgG1 and IgG3 levels increased after the second week of illness. The median IgG1 and IgG3 levels in the acute serum samples were 1.08 (IQR = 0.97–1.31) and 1.03 (IQR = 1.01–1.18), respectively. The median levels of IgG1 and IgG3 at the convalescent period increased to 1.62 (IQR = 1.28–2.19) and 1.96 (IQR = 1.17–2.82), respectively (both *P* = 0.013). RBD-specific IgG1 was detected in nine of ten patients (90%) except P5 and IgG3 was detected in eight of ten patients (80%) except P5 and P10 after the second week of illness. Four patients (P6, P9, P12 and P13) showed elevated both IgG1 and IgG3 levels more than 1.5-fold at the convalescent period. In contrast, the median levels of IgG2 and IgG4 did not increase significantly between the admission and convalescent period.

We plotted the correlations between levels of total IgG and IgG subclasses in COVID-19 patients (S2 Fig). There was a moderate correlation between total IgG and IgG1 levels (rho = 0.65, *P* < 0.001, S2A Fig). The IgG3 level showed a high correlation with total IgG level (rho = 0.86, *P* < 0.001, S2C Fig). We observed no correlation between total IgG level and IgG2 level (rho = 0.13, *P* = 0.52, S2B Fig) nor between total IgG level and IgG4 level (rho = -0.14, *P* = 0.48, S2D Fig).

We further analyzed the association of IgG subclasses with the characteristics of the COVID-19 patients (S2 Table). The SARS-CoV-2 specific IgG1, IgG2, IgG3 and IgG4 antibodies were detected in 12, 2, 10 and 4 patients, respectively. Age and sex were not associated with IgG subclass detection. But pneumonia was associated with high IgG4 levels (*P* = 0.033; S2 Table). The median duration of earliest IgG1, IgG2, IgG3 and IgG4 antibody detection was 6 days (IQR = 3.5–20.5), 16.5 days (IQR = 4–29), 14 days (IQR = 4–21) and 3.5 days (IQR = 0–8.5) after diagnosis, respectively. Four of six patients (P4, P5, P7 and P10) with undetectable total IgG had at least one IgG subclass.

### Correlation between RBD-specific antibodies level and Ct values of *ORF1ab* gene in COVID-19 patients

We next determined the association between of antibody response and viral load during diagnosis in COVID-19 patients. We analyzed the correlation between RBD-specific antibodies level and Ct values of *ORF1ab* gene of SARS-CoV-2 (S3 and S4 Figs). For both acute and convalescent-phase serum samples N = 10 and 12, respectively), there were no significant correlations between the levels of antibody classes and Ct values of the *ORF1ab* gene (S3A–S3E Fig) except a low negative correlation in the levels of total IgG of convalescent serum with the Ct-*ORF1ab* gene (rho = -0.46, S3F Fig).

The correlation between levels of IgG subclasses and Ct values of *ORF1ab* gene in COVID-19 patients in the acute serum samples and the convalescent serum samples is shown in S4 Fig. The Ct-*ORF1ab* gene showed a moderate negative correlation with the IgG3 levels (rho = -0.57, *P* = 0.08, S4C Fig) in the acute serum samples. The results from the acute serum samples indicated a low correlation in levels of IgG1, IgG2 and IgG4 with the Ct-*ORF1ab* gene (rho = 0.44, -0.47, 0.34, S4A, S4B and S4D Fig, respectively). The Ct-*ORF1ab* gene was low correlated to IgG2 and IgG4 subclasses in the convalescent serum samples (rho = -0.40 and 0.48; S4F and S4H Fig).

### Antibody response to RBD of SARS-CoV-2 among patients with other infections and healthy individuals

**Fig 3:**
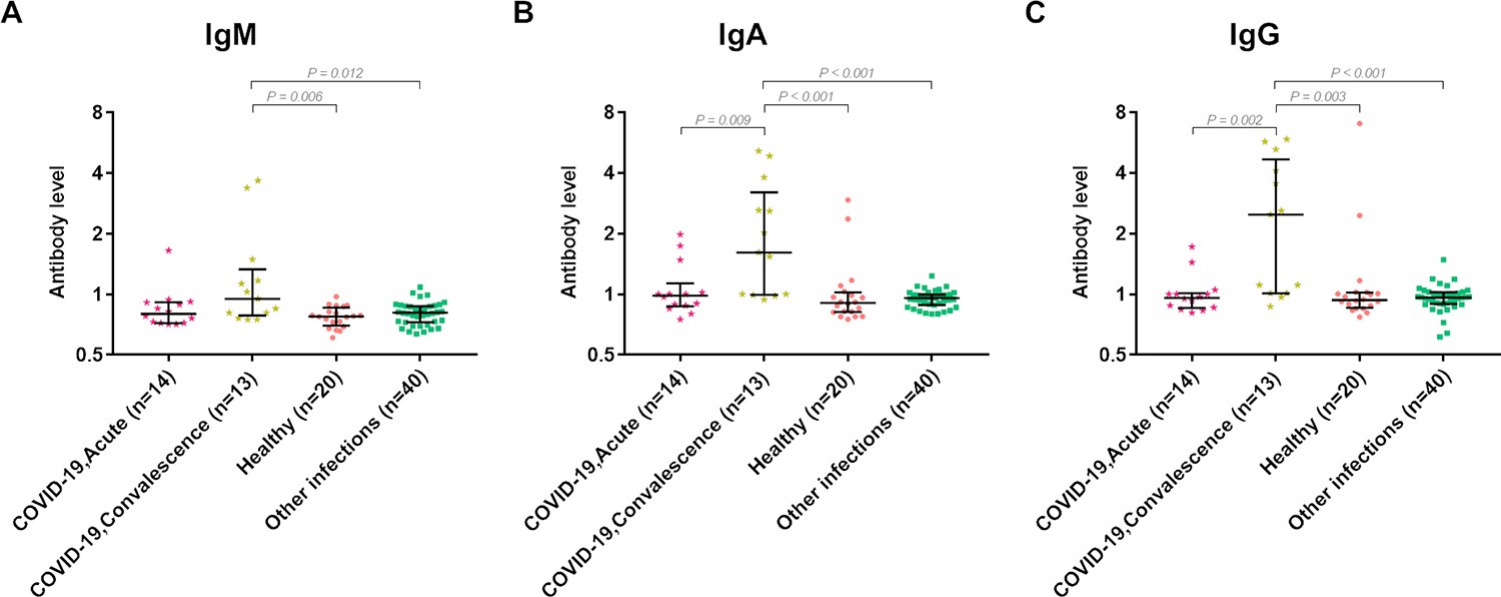
Analysis of classes of antibody responses to RBD in serum samples of acute and convalescent-phase COVID-19 patients, healthy donors and patients with other infections.

We used the serum of patients with other infectious diseases before the COVID-19 pandemic and healthy subjects as negative controls. We compared the antibody response to RBD in the acute COVID-19 samples and convalescent COVID-19 samples with the convalescent serum of patients with other infections and healthy subjects (Fig 3).

The median IgM level in the convalescent-phase serum samples of COVID-19 patients (0.95, IQR = 0.81–1.17) was significantly higher than in healthy donors (0.78, IQR = 0.71–0.85) and patients with other infectious disease (0.81, IQR = 0.73–0.87, both *P* < 0.05, Fig 3A). There were no significant differences in the median IgM level of acute serum samples of COVID-19 patients with convalescent-phase serum samples of COVID-19 patients or serum samples of patients with other infections or healthy donors (Fig 3A).

The median IgA level in the convalescent-phase serum samples increased approximately twofold (1.61, IQR = 1.00–2.61) and significantly higher than the acute serum samples (0.98, IQR = 0.88–1.02) of COVID-19 patients (*P* = 0.009, Fig 3B). The median IgA levels in the serum samples of healthy controls and patients with other infectious diseases were 0.91 (IQR = 0.82–1.01) and 0.96 (IQR = 0.89–1.0), respectively. The median level of IgA in the acute serum samples of COVID-19 patients was not significantly different from the serum samples of patients with other infections. However, the median IgA level in convalescent serum samples was significantly higher compared to the serum samples of healthy donors and those of patients with other infections (both *P* < 0.001, Fig 3B).

The median level of IgG in the convalescent-phase serum samples was 2.6-fold higher (2.48, IQR = 1.01–4.1) than in the acute serum samples (0.97, IQR = 0.86–1.00) of COVID-19 patients (*P* = 0.002, Fig 3C). The median IgG levels in serum samples of healthy controls and patients with other infectious diseases were 0.94 (IQR = 0.86–1.02) and 0.96 (IQR = 0.90–1.02), respectively. The median IgG level in convalescent serum of COVID-19 patients was significantly higher than in the serum samples of healthy donors and patients with other infections (*P* = 0.003 and *P* < 0.001, respectively, Fig 3C).

### Performance of specific antibodies in discriminating patients with COVID-19 and other infections

We evaluated the diagnostic role of these antibody detection assay in COVID-19 infection (Table 2). Fourteen samples of acute samples and 13 samples of convalescent serum were analyzed with 60 samples of pre-COVID-19 pandemic including 20 samples from healthy donors and 40 samples from others tropical infectious diseases. Using threshold ratio of this assay as 1.05, the detection of IgG antibody level in convalescent serum showed highest sensitivity (69.23%) followed by convalescent IgA 61.54% sensitivity. The detection of IgM level in serum showed highest specificity (98.33%) but poor sensitivity (7.14% for acute serum and 38.46% for convalescent serum).

**Table 2:** Sensitivity, specificity, positive predictive value and negative predictive value of RBD-specific antibody detection by ELISA test.

Antibody	Sample	Sensitivity (%)	Specificity (%)	Positive predictive value (%)	Negative predictive value (%)
IgM	Acute	7.14	98.33	50.00	81.94
	Convalescent	38.46	98.33	83.33	88.06
IgA	Acute	21.43	85.00	25.00	82.26
	Convalescent	61.54	85.00	47.06	91.07
IgG	Acute	21.43	81.67	21.43	81.67
	Convalescent	69.23	81.67	45.00	92.45

**Fig 4:**
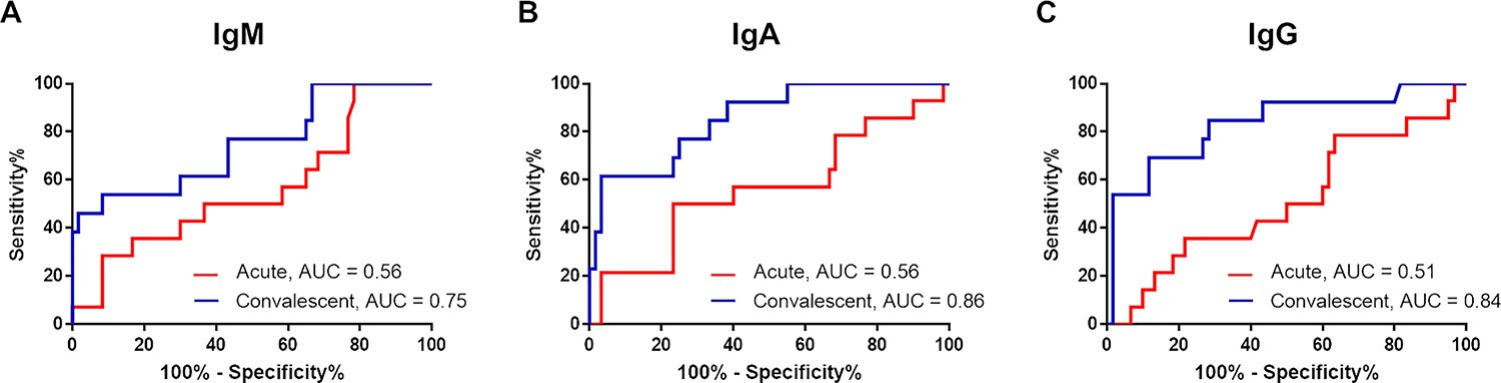
Receiver operating characteristics (ROC) curves of specific antibodies obtained from the acute and convalescent serum of COVID-19 patients. The area under the ROC curve (AUC) is shown for IgM (A), IgA (B) and IgG (C).

ROC analysis was performed to evaluate suitable threshold ratio of the assay. The area under ROC curves (AUC) was calculated for IgM, IgA and IgG antibodies during the acute and convalescent phases (Fig 4). The AUC for acute phase was 0.56 (95% CI 0.39–0.74) for IgM, 0.56 (95% CI 0.38–0.74) for IgA and 0.51 (95% CI 0.34–0.69) for IgG. The AUC for convalescent phase was 0.75 (95% CI 0.59–0.91) for IgM, 0.86 (95% CI 0.75–0.96) for IgA and 0.84 (95% CI 0.70–0.97) for IgG (Fig 4A–4C).

The ROC analysis revealed the enhanced sensitivity of IgA and IgG antibody response in the convalescent phase when modified the threshold ratio of the assay. The sensitivity of IgA antibody detection increased to 76.92% (95% CI = 46.19% - 94.96%) but specificity dropped to 75% (95% CI = 62.14% - 85.28%) at cut-off value of 0.99. The specificity of IgG antibody improved to 76.92% (95% CI = 46.19% - 94.96%) but specificity reduced to 71.67% (95% CI = 58.56–82.55%) at cut-off value of 1.008.

## Discussion

We have developed ELISAs for IgM, IgA, IgG and IgG subclasses of antibodies against RBD antigen of SARS-CoV-2. Our analyses revealed that 26.67% of COVID-19 patients had detectable IgM levels while 53.33–60% had specific IgA and IgG levels during the study period. 80% of patients during the convalescent period showed increased levels of antibodies but 20% of pateints who had paired sera showed no antibody responses over time. We observed the highest correlation between levels of IgG and IgA (rho = 0.92) followed by between levels of IgA and IgM (rho = 0.87). IgG1 and IgG3 were the major IgG subclasses of total IgG responding to SARS-CoV-2 infection. Furthermore, IgG3 level at admission was negatively associated with viral load based on RT-PCR of *ORF1ab* gene (rho = -0.57). The median IgA and IgG levels in convalescence sera of COVID-19 patients were significantly higher than healthy individuals and non-COVID-19 febrile patients. The analyses of antibody classes and subclasses provide insights into human immune responses against SARS-CoV-2 during natural infection and interpretation of antibody assays.

Recent studies in the USA and Canada reported that RBD is a highly immunogenic antigen [27, 28]. They revealed that specific IgM, IgG and IgA against RBD of SARS-CoV-2 can be detected by ELISA in serum or plasma samples of COVID-19 patients [27, 28]. From the analysis of 348 SARS-CoV-2 infected patients in Northern America and 1,548 blood samples collected prior COVID-19 outbreak, RBD-based ELISA had a high sensitivity of 95% for IgG, 90% for IgA and 81% for IgM for detecting infected individuals [27]. The study in Canada also showed 94% sensitivity of RBD-based ELSA for detection of IgG in serum from evaluation of 402 PCR-confirmed COVID-19 samples and 399 banked pre-COVID samples [28]. The specific antibodies against RBD increased after 1–3 weeks of symptoms onset [27, 28]. We also detected the high level of IgA and IgG antibodies in many COVID-19 patients, except IgM, although the assays were performed in convalescent serum samples at 3 weeks after diagnosis by RT-PCR. Sensitivity and specificity of our assay were 61.5% and 85% for convalescent IgA and 69.2% and 85% for convalescent IgG. The discrepancy in the results between our study and those studies may be due to the difference in the severity of COVID-19, population and different cut-off value of the assays.

Other studies also showed that the level of IgG antibody positively relates to the severity of the disease [14, 15, 29–31]. The severe COVID-19 patients in Belgium had a high level of neutralizing antibody than mild cases [29]. The asymptomatic cases had IgG antibody response lower than patients who had a symptom of respiratory tract infection [30]. Moreover, the COVID-19 patients with pneumonia and hypoxia had a high level of spike1-specific IgA and IgG than mild and moderate cases [31]. The children with the severe multisystem inflammatory syndrome in children (MIS-C) had IgM and IgG against RBD of the spike of SARS-CoV-2; however, IgM and IgG-specific antibodies were not detectable in mild MIS-C cases [14, 15]. Our result also revealed that the IgM, IgA, IgG, IgG1 and IgG3 antibody levels of patients with pneumonia (P1, P9, P12 and P13) were higher than mild cases at the same period. Patient no.9 (P9) had pneumonia and other underlying diseases such as arterial hypertension, obesity and diabetes mellitus that are important associated with severe symptoms in COVID-19 disease [32]. The combination of these factors might contribute to such a high induction of antibody response to RBD of SARS-CoV-2 in this patient.

Recent studies revealed that IgG was more stable than IgA and IgM [27–29]. IgM and IgA have a short-life while IgG antibody is stable for more than three months [27, 28]. Additionally, 96% of COVID-19 patients had detectable IgG levels up to five months after infection [29]. In this study, specific IgM was detected in only four cases who had pneumonia symptoms. The mild COVID-19 cases in this study might have a lower amount of IgM with rapid decay leading to a lower IgM level than our assay threshold.

There is evidence that the time of specific antibody development is associated with the severity of COVID-19. The median time of IgG antibody response in severe cases (11 days) was shorter than in mild cases (22 days) [33]. Our study also found that the RBD-specific antibodies were detected after 2 weeks of illness. The patients with pneumonia showed specific IgA earlier than patients with mild symptoms.

Previous studies in patients with HIV and HCV infections reported that IgG3 was associated with viral clearance and neutralization [18, 19]. The IgG1 and IgG3 were detected after the peak of HCV viral load where the HCV-infected clearers developed antibodies early and at a higher level than the chronic patients [19]. This study also found SARS-CoV-2 RBD-specific IgG1 and IgG3 antibodies in COVID-19 patients and a high level of IgG3 was correlated with the low Ct value of the *ORF1ab* gene (high SARS-CoV-2 viral load). Measurements of IgG3 level might be useful for the determination of the SARS-CoV-2 viral clearance and IgG3 induction by vaccination. However, the dynamic response of IgG3 in SARS-CoV-2 infection requires further evaluation.

Several studies reported that a few COVID-19 patients had undetectable specific antibody response to SARS-CoV-2 spike and nucleocapsid [29, 34], however, neutralizing antibody was detectable using a neutralizing antibody assay with Vero cells [33]. Our study also observed the non-antibody responders for RBD antigen for all antibody classes and subclasses in 13.33% (2 of 15) of COVID-19 patients. Both patients had mild symptoms and their samples were collected once after admission. One of them (Patient: P3) was questionable in COVID-19 diagnosis due to a no exposure history, weak positive RT-PCR at diagnosis and negative COVID-19 test results in the next 3 days. Unfortunately, the first respiratory specimen of this case was not available for confirmation. Another non-antibody responder had a history of alcoholism.

Other tropical diseases, such as dengue, murine typhus, seasonal influenza and leptospirosis are major causes of febrile illness in Thailand [22]. Both RBD-specific IgA and IgG antibodies can be used to distinguish COVID-19 from other these tropical infectious diseases in the convalescent phase of the disease. Our ROC curve analysis revealed that the IgA and IgG antibody levels of specific antibodies against RBD of SARS-CoV-2 may be useful for serological diagnosis of COVID-19 patients in the convalescent period when the molecular methods have a limited diagnostic role in all infectious diseases including SARS-CoV-2. Additionally, specific antibody detection may be an advantage for diagnosis in complicated cases such as MIS-C in children who have low viral load but produce detectable levels of specific IgM and IgG antibodies [14, 15].

Recently, several COVID-19 vaccines are available. Major of them induce antibody response to spike protein of SAR-CoV-2 by introducing recombinant spike protein (eg. NVX-CoV2373, ZF2001), replication-incompetent adenovirus vector encoding spike protein (eg. ChAdOx1 nCoV-19, Ad26.COV2.S and rAd26-S+rAd5-S), mRNA encoding spike protein (BNT162b2 and mRNA-1273) or inactivated SARS-CoV-2 (CoronaVac and BBIBP-CorV). These antibody responses, developed in vaccinated subjects have been shown to be correlated with neutralizing antibody [35–42]. Our ELISA assay may be used for monitoring classes and subclasses of antibody response to spike protein after vaccination. However, is not clear what classes and subclasses of antibody response being predominant in vaccinated individuals by various types of vaccine. The profile of antibody response to vaccine might be different form natural infection with SAR-CoV-2 variants. This subject needs more investigation.

Our study has some limitations. First, the sample size of the COVID-19 cohort in the hospital is small during the first outbreak in Thailand and may limit data interpretation. Second, race, age and distribution of other infectious diseases in this study may limit the generalization and relevance of the results in other settings.

In conclusion, this study demonstrated that IgA, total IgG, IgG1 and IgG3 are the major antibody responses against RBD of the spike of SARS-CoV-2 in COVID-19 patients. The RBD-specific antibody response in convalescent samples is specific to SARS-CoV-2 infection which differs from other tropical infectious diseases. Our results suggest that the RBD-specific antibody detection is a potential assay for the examination of the immune response in SARS-CoV-2 infection and vaccination. Therefore, serological assays to detect antibodies should be useful for diagnosis at later phases of infection, epidemiological study and evaluation of immunization.

## Supporting information

S1 Fig Correlation between RBD-specific IgM, IgA and IgG levels in COVID-19 patients. Correlation of antibody response of total IgM versus total IgA (A), total IgG versus total IgM (B) and total IgG versus total IgA (C).
(TIF)

S2 Fig Correlation between different IgG subclasses of antibody responses to RBD in COVID-19 patients. Correlation of antibody response of total IgG versus IgG1 (A), IgG2 (B), IgG3 (C) and IgG4 (D).
(TIF)

S3 Fig The correlation between RBD-specific antibodies level and Ct values of *ORF1ab* gene in COVID-19 patients. Correlation of Ct-*ORF1ab* with antibody levels at acute phase is shown in A-C and at convalescent-phase is shown in D-F.
(TIF)

S4 Fig Correlation between different IgG subclasses of antibody responses to RBD and Ct values (*ORF1ab* gene) of COVID-19 patients. Correlation of Ct-*ORF1ab* with antibody levels at acute phase (A-D) and convalescent-phase (E-H).
(TIF)

S1 Table Data of IgM, IgA and IgG in each COVID-19 patient. (PDF)

S2 Table Characteristics of COVID-19 patients and classes and subclasses of antibody response against RBD of SARS-CoV-2. (PDF)

S3 Table Data of IgG1, IgG2, IgG3 and IgG4 in each COVID-19 patient. (PDF)
