# Supplementary figures and images for: Longitudinal analysis to characterize classes and subclasses of antibody responses to recombinant receptor-binding protein (RBD) of SARS-CoV-2 in COVID-19 patients in Thailand
Source: PLoS One. 2021 Aug 10;16(8):e0255796. doi: 10.1371/journal.pone.0255796 (PMC8354433; doi:10.1371/journal.pone.0255796)

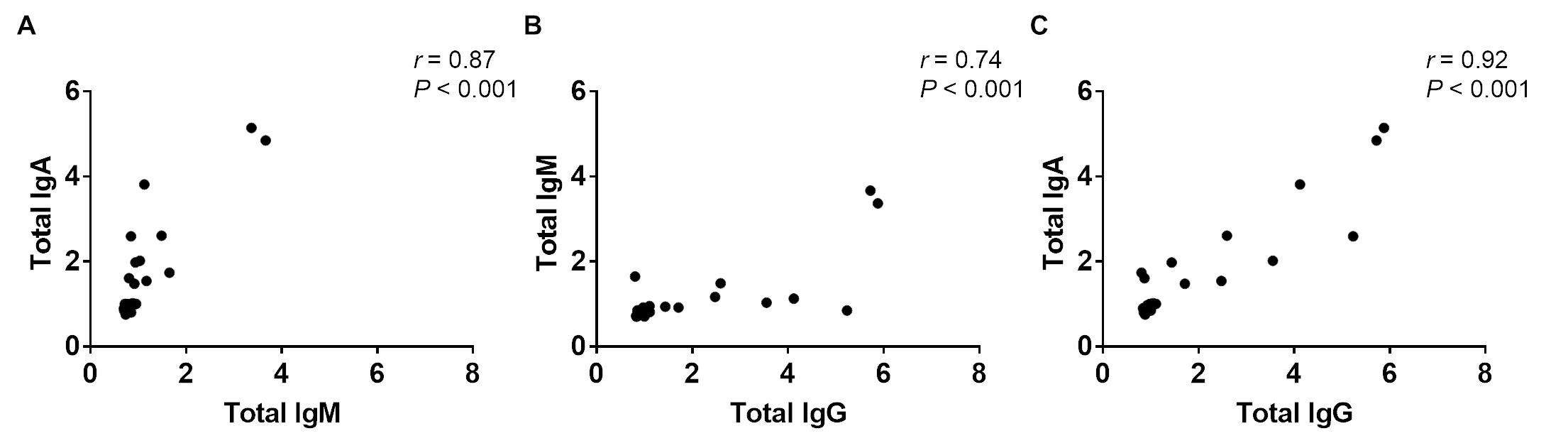

Supplement: S1 Fig — Correlation of antibody response of total IgM versus total IgA (A), total IgG versus total IgM (B) and total IgG versus total IgA (C). (TIF) [file pone.0255796.s001.tif]

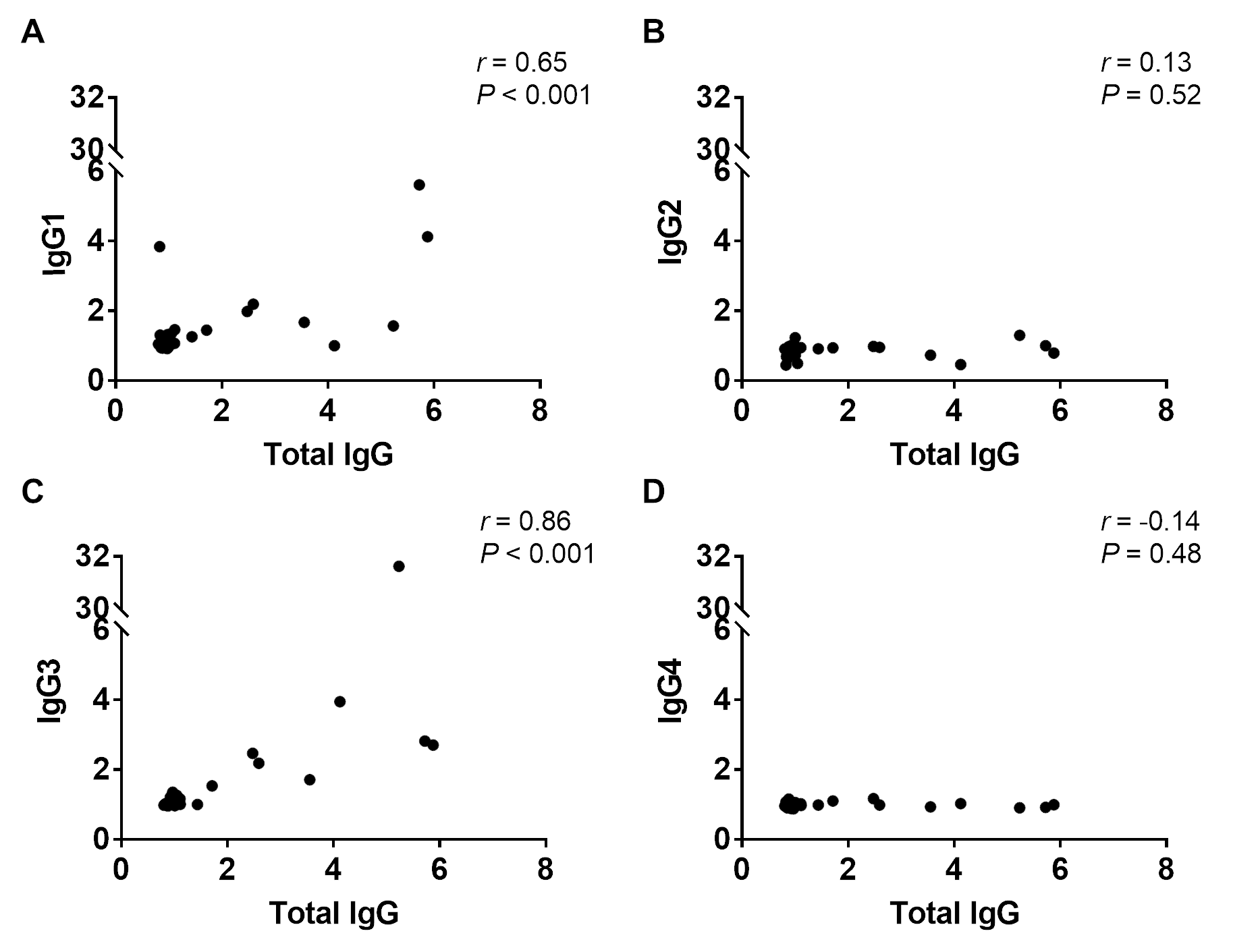

Supplement: S2 Fig — Correlation of antibody response of total IgG versus IgG1 (A), IgG2 (B), IgG3 (C) and IgG4 (D). (TIF) [file pone.0255796.s002.tif]

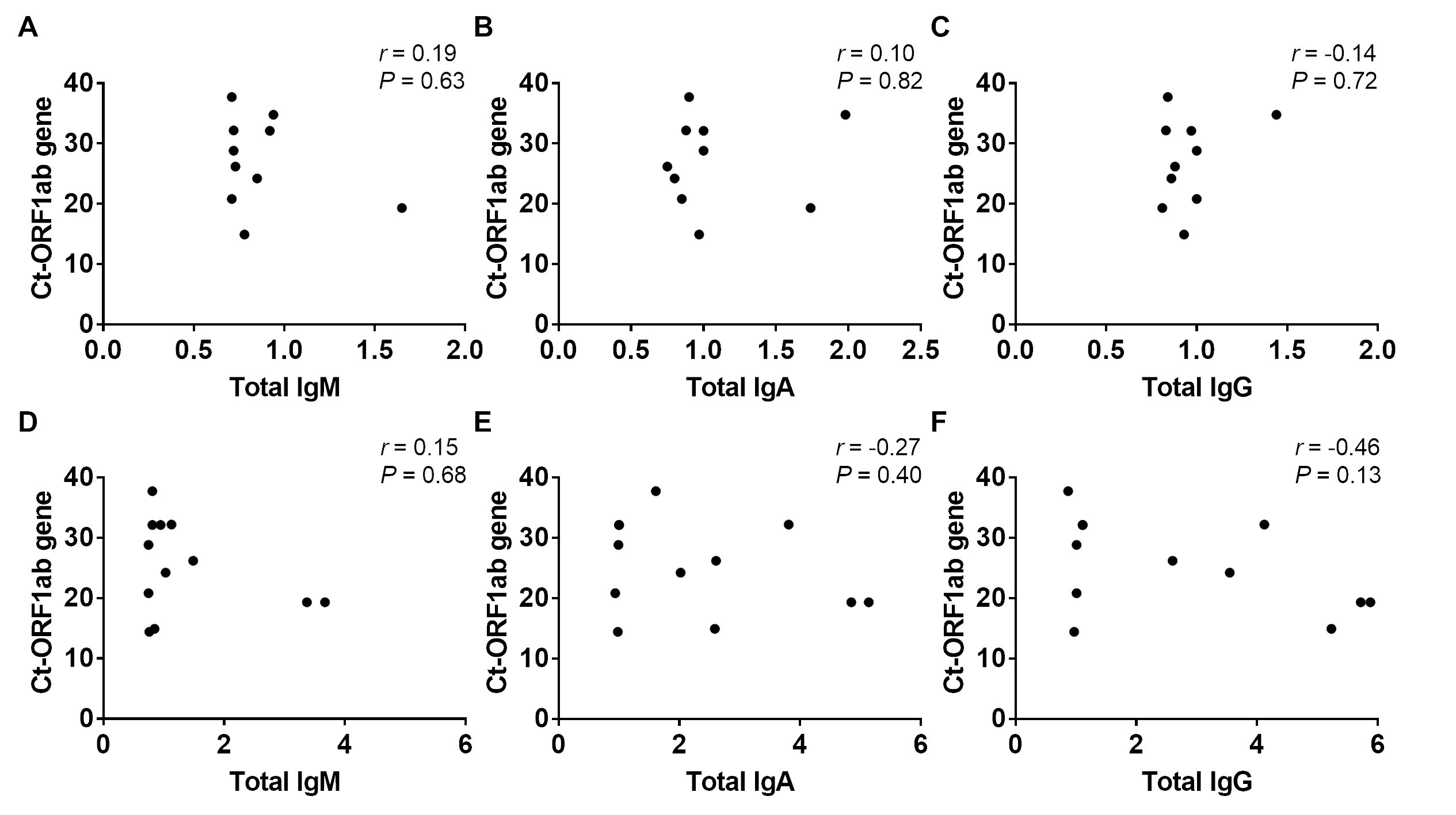

Supplement: S3 Fig — Correlation of Ct-ORF1ab with antibody levels at acute phase is shown in A-C and at convalescent-phase is shown in D-F. (TIF) [file pone.0255796.s003.tif]

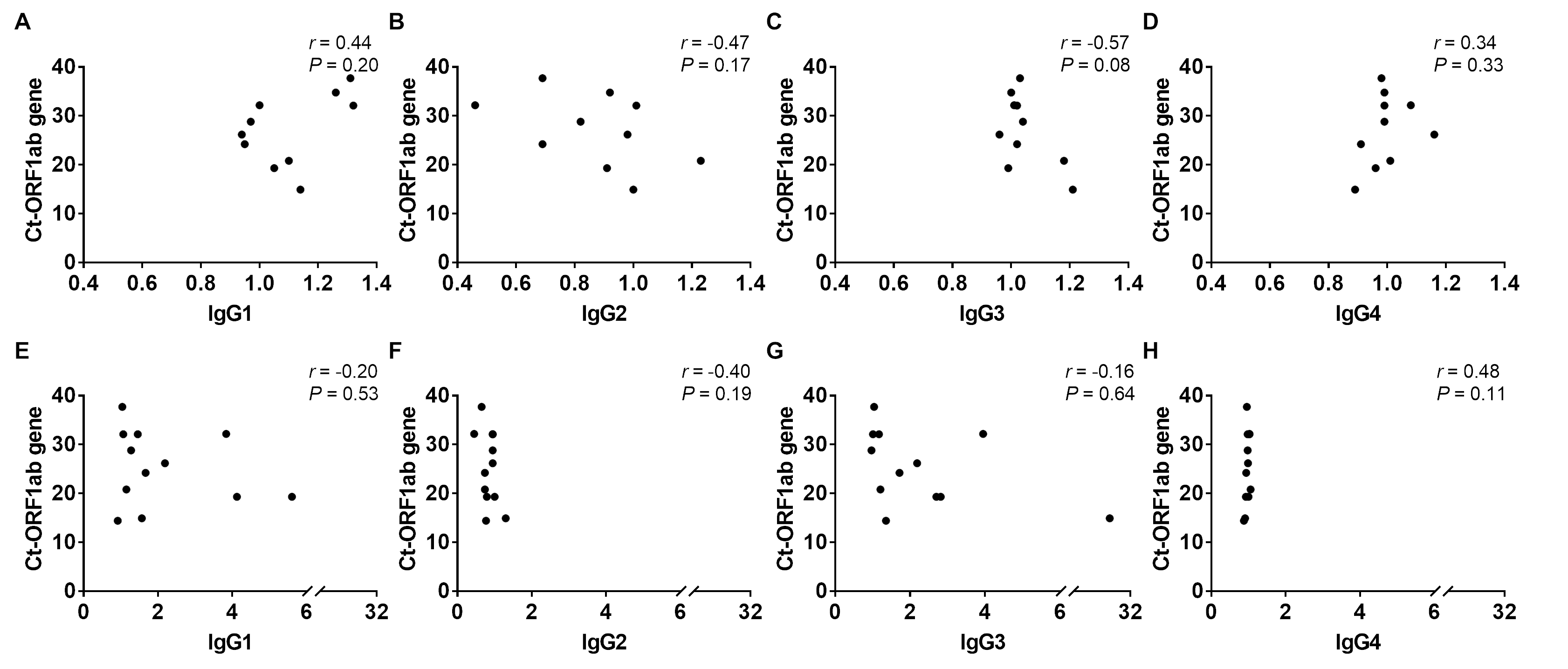

Supplement: S4 Fig — Correlation of Ct-ORF1ab with antibody levels at acute phase (A-D) and convalescent-phase (E-H). (TIF) [file pone.0255796.s004.tif]
