# Supplementary material for: Longitudinal analysis to characterize classes and subclasses of antibody responses to recombinant receptor-binding protein (RBD) of SARS-CoV-2 in COVID-19 patients in Thailand
Source: PLoS One. 2021 Aug 10;16(8):e0255796. doi: 10.1371/journal.pone.0255796 (PMC8354433; doi:10.1371/journal.pone.0255796)
Supplement: S1 Table — (PDF) [file pone.0255796.s005.pdf]

S1 Table. Data of IgM, IgA and IgG in each COVID-19 patient

Patients	Antibody level					
	IgM		IgA		IgG	
	Acute serum	Convalescent serum	Acute serum	Convalescent serum	Acute serum	Convalescent serum
**P1**	0.91	1.17	1.48	1.54	1.72	2.48
**P2**	0.78	0.85	0.97	2.59	0.93	5.23
**P3**	0.76	NA	1.00	NA	1.00	NA
**P4**	0.71	0.75	0.85	0.94	1.00	1.01
**P5**	0.71	0.81	0.90	1.61	0.84	0.87
**P6**	0.85	1.03	0.80	2.02	0.86	3.55
**P7**	NA	0.76	NA	0.98	NA	0.97
**P8**	0.92	0.95	1.00	1.00	0.97	1.11
**P9**	1.65	3.67	1.74	5.14	0.81	5.88
**P10**	0.72	0.75	1.00	0.99	1.00	1.01
**P11**	0.82	NA	0.89	NA	0.95	NA
**P12**	0.73	1.49	0.75	2.61	0.88	2.60
**P13**	0.72	1.13	0.88	3.81	0.83	4.12
**P14**	0.89	NA	1.02	NA	1.05	NA
**P15**	0.94	NA	1.98	NA	1.44	NA
