# Supplementary material for: Longitudinal analysis to characterize classes and subclasses of antibody responses to recombinant receptor-binding protein (RBD) of SARS-CoV-2 in COVID-19 patients in Thailand
Source: PLoS One. 2021 Aug 10;16(8):e0255796. doi: 10.1371/journal.pone.0255796 (PMC8354433; doi:10.1371/journal.pone.0255796)
Supplement: S2 Table — (PDF) [file pone.0255796.s006.pdf]

S2 Table. Characteristics of COVID-19 patients and classes and subclasses of antibody response against RBD of SARS-CoV-2

Characteristics	Antibody						
	IgM (n = 4)	IgA (n = 8)	IgG (n = 9)	IgG1 (n = 12)	IgG2 (n = 2)	IgG3 (n = 10)	IgG4 (n = 4)
**Age**	51.5	37.5	36	33	40.5	36	41.5
**(IQR)**	(38.5 - 62)	(27.5 - 51.5)	(27 - 47)	(27.5-46)	(36 - 45)	(27 - 47)	(33 - 51.5)
***P*-value**	0.019	0.246	0.215	0.148	0.268	0.197	0.077
**Male (n = 8; (%))**	3 (75.0)	5 (62.5)	6 (66.7)	7 (58.3)	1 (50.0)	5 (50.0)	2 (50.0)
**Female (n = 7; (%))**	1 (25.0)	4 (37.5)	3 (33.3)	4 (41.7)	1 (50.0)	5 (50.0)	2 (50.0)
***P*-value**	0.338	0.405	0.231	0.446	0.733	0.573	0.662
**Pneumonia (n = 4 (%))**	4 (100)	4 (50.0)	4 (44.4)	4 (33.3)	0 (0)	4 (40.0)	3 (75.0)
***P*-value**	0.001	0.051	0.092	0.363	0.524	0.154	0.033
