# Supplementary material for: Longitudinal analysis to characterize classes and subclasses of antibody responses to recombinant receptor-binding protein (RBD) of SARS-CoV-2 in COVID-19 patients in Thailand
Source: PLoS One. 2021 Aug 10;16(8):e0255796. doi: 10.1371/journal.pone.0255796 (PMC8354433; doi:10.1371/journal.pone.0255796)
Supplement: S3 Table — (PDF) [file pone.0255796.s007.pdf]

## S3 Table. Data of IgG1, IgG2, IgG3 and IgG4 in each COVID-19 patient

| Patient | Antibody level | | | | | | | |
|---|---|---|---|---|---|---|---|---|
| | IgG1 | | IgG2 | | IgG3 | | IgG4 | |
| | Acute serum | Convalescent serum | Acute serum | Convalescent serum | Acute serum | Convalescent serum | Acute serum | Convalescent serum |
| P1 | 1.45 | 1.99 | 0.94 | 0.98 | 1.54 | 2.47 | 1.10 | 1.17 |
| P2 | 1.14 | 1.57 | 1.00 | 1.30 | 1.21 | 31.63 | 0.89 | 0.91 |
| P3 | 0.99 | NA | 1.00 | NA | 1.04 | NA | 1.01 | NA |
| P4 | 1.10 | 1.15 | 1.23 | 0.74 | 1.18 | 1.21 | 1.01 | 1.06 |
| P5 | 1.31 | 1.04 | 0.69 | 0.65 | 1.03 | 1.04 | 0.98 | 0.96 |
| P6 | 0.95 | 1.67 | 0.69 | 0.74 | 1.02 | 1.72 | 0.91 | 0.94 |
| P7 | NA | 0.92 | NA | 0.77 | NA | 1.36 | NA | 0.88 |
| P8 | 1.32 | 1.46 | 1.01 | 0.95 | 1.02 | 1.17 | 0.99 | 1.03 |
| P9 | 1.05 | 5.61 | 0.91 | 1.00 | 0.99 | 2.82 | 0.96 | 1.00 |
| P10 | 0.97 | 1.28 | 0.82 | 0.95 | 1.04 | 0.97 | 0.99 | 0.98 |
| P11 | 1.03 | NA | 0.93 | NA | 1.01 | NA | 0.99 | NA |
| P12 | 0.94 | 2.19 | 0.98 | 0.95 | 0.96 | 2.19 | 1.16 | 0.99 |
| P13 | 1.00 | 3.84 | 0.46 | 0.45 | 1.01 | 3.95 | 1.08 | 1.03 |
| P14 | 1.35 | NA | 0.50 | NA | 1.27 | NA | 1.00 | NA |
| P15 | 1.26 | NA | 0.92 | NA | 1.00 | NA | 0.99 | NA |
